# Hesperidin-Loaded Lipid Polymer Hybrid Nanoparticles for Topical Delivery of Bioactive Drugs

**DOI:** 10.3390/ph15020211

**Published:** 2022-02-10

**Authors:** Rajendra Jangde, Gamal Osman Elhassan, Sulekha Khute, Deependra Singh, Manju Singh, Ram Kumar Sahu, Jiyauddin Khan

**Affiliations:** 1 University Institute of Pharmacy, Pt. Ravishankar Shukla University, Raipur 492010, India; sulekhakhunte@gmail.com (S.K.); deependraiop@gmail.com (D.S.); manjursu@gmail.com (M.S.); 2 Department of Pharmaceutics, Unaizah College of Pharmacy, Qassim University, Unaizah 51911, Saudi Arabia; go.osman@qu.edu.sa; 3 Department of Pharmaceutical Sciences, Assam University (A Central University), Silchar 788011, India; 4 School of Pharmacy, Management & Science University, Shah Alam 40100, Selangor Darul Ehsan, Malaysia

**Keywords:** hesperidin, hybrid nanoparticles, emulsion solvent evaporation, DPPH model, sustained release

## Abstract

Hesperidin is a bioflavonoid constituent that among many other biological activities shows significant wound healing properties. However, the bioavailability of hesperidin when applied topically is limited due to its low solubility and systemic absorption, so novel dosage forms are needed to improve its therapeutic efficacy. The objectives of this study were to develop hesperidin-loaded lipid-polymer hybrid nanoparticles (HLPHNs) to enhance the delivery of hesperidin to endogenous sites in the wound bed and promote the efficacy of hesperidin. HLPHNs were optimized by response surface methodology (RSM) using the Box-Behnken design. HLPHNs were prepared using an emulsion-solvent evaporation method based on a double emulsion of water-in-oil-in-water (w/o/w) followed by freeze-drying to obtain nanoparticles. The prepared formulations were characterized using various evaluation parameters. In addition, the antioxidant activity of HLPHN 4 was investigated in vitro using the DPPH model. Seventeen different HLPHNs were prepared and the HLPHN4 exhibited the best mean particle size distribution, zeta potential, drug release and entrapment efficiency. The values are 91.43 nm, +23 mV, 79.97% and 92.8%, respectively. Transmission electron microscope showed similar spherical morphology as HLPHN4. Differential scanning calorimetry verified the physical stability of the loaded drug in a hybrid system. In vitro release studies showed uniform release of the drug over 24 h. HLPHN4 showed potent antioxidant activity in vitro in the DPPH model. The results of this study suggest that HLPHNs can achieve sustained release of the drug at the wound site and exhibit potent in vitro antioxidant activity.

## 1. Introduction

**Figure 1:**
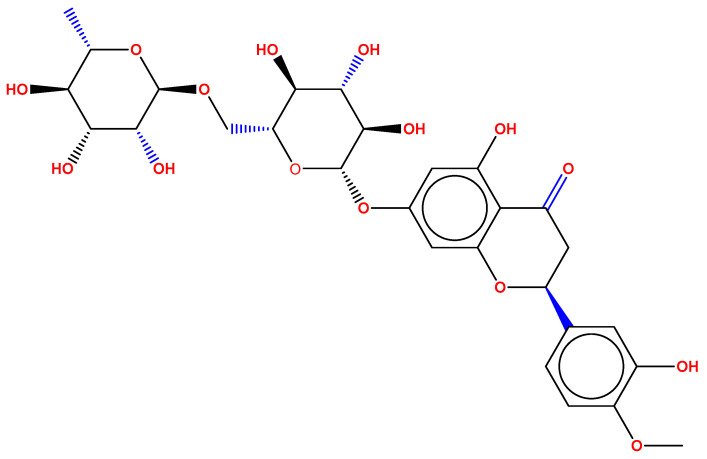
Structure of hesperidin.

Hesperidin (Figure 1) is a bioflavonoid mainly extracted from various citrus fruits. Numerous researchers have found that hesperidin has antioxidant properties and additionally possesses antitumor, antimicrobial, anti-inflammatory, antidiabetic and hepatoprotective effects [1,2]. The significant wound-healing effect of hesperidin has been demonstrated in animal models of excision wound healing and in clinical studies. In addition, hesperidin also reduces the formation of fibroses and scars after wound healing in experimental animals. Hesperidin is used to treat tropical infections by placing a dressing over them. Treatment of wounds with drugs can lead to undesirable side effects on healthy cells due to systemic delivery of drugs and also decreases the bioavailability of drugs [3,4,5]. Accordingly, several studies have focused on the local delivery of the drug for the healing process of the wound through the dressing. This could avoid adverse effects on non-target tissues and deliver drugs directly to the target tissue. Therefore, it is very important to improve the delivery of the topical formulations containing hesperidin without adverse effects and increase the bioavailability of the drug.

Nanotechnology is a remarkable medical platform, especially for its potential to significantly impact the delivery of a wide range of therapeutic drugs, including small molecule curative agents, RNAs, genes, peptides, and diagnostic imaging agents, as well as a compelling promise to improve the therapeutic index and pharmacokinetics of various drugs in systemic systems. Despite these advantages, nanotechnology has certain limitations, such as lower dispersibility in the aqueous phase and poor solubility. Therefore, by integrating polymeric and lipid-based nanocarriers in the form of lipid-polymer hybrid nanoparticles (LPHNPs), nanotechnology has introduced a unique tool to overcome the potential shortcomings of polymeric nanoparticles and lipid nanocarriers.

The lipid-polymer hybrid system, which enables slow drug diffusion, improves drug loading, avoids non-specific release, minimizes dose-dependent toxicity, enhances stability and allows controlled drug delivery [6]. Xiao et al., reported lower therapeutic efficacy and faster drug release of highly hydrophilic drugs, as these drugs are associated with rapid excretion in the body and poor bioavailability. In the hybrid system, the drug is conjugated with a target receptor affinity to deliver the drug to its target site in a controlled manner [7]. Dave et al., developed LPHNPs as a suitable carrier for safe, effective and better therapeutic efficacy of norfloxacin for the treatment of bacterial infections [8]. Chitosan is indeed a great natural polymer because of its eco-friendly, hemostatic and biocomplex properties, but it does not have enough elasticity to break into simple small pieces [9]. Although the chitosan polymer is used in the healing process to repair wounds, it is desirable to promote the appropriate prevention of skin injuries [10]. Due to its versatile physical properties, such as expansion and degradability, chitosan can control the release rate of drugs, which makes it a useful tool for clinical use. Hesperidin has a stronger antioxidant and anti-inflammatory effect on the collagen in the wound area, improving the fusion process. Therefore, it is believed that a mixture of chitosan and hesperidin may have a synergistic effect on rapid wound healing [11]. Nanoparticles loaded with hesperidin have not yet been investigated in wound healing research. Therefore, in the present work, hesperidin-loaded nanoparticles were characterized by a 3D plane of factorial design software. The optimized formula is subjected to a scale-up process. Hesperidin has lower bioavailability with increased protein binding and rapid excretion. The bioavailability and therapeutic value of hesperidin could be increased by the sustained release formulation.

In this study, the Box-Behnken design method was used to observe the conditions and analyze the sensitivity of HLPHNs at endogenous sites in the wound bed. HLPHNs were optimized using response surface methodology (RSM). HLPHNs were prepared using the emulsion-solvent evaporation technique based on a water-in-oil-in-water (w/o/w) double emulsion followed by freeze-drying to recover the nanoparticles. PDI, FTIR, DSC, XRD and TEM were used in this work to characterize the physical and chemical properties of HLPHNs.

## 2. Results and Discussions

### 2.1. Optimization of HLPHNs

Different batches of HLPHNs were prepared using an emulsion solvent evaporation method and the effects of lipid concentration and drug:polymer ratio were evaluated. This is a simple and effective way of preparing HLPHNs in the laboratory. The HLPHNs were acceptable based on the results of the quadratic response surface. The optimal formulation variables selected were, i.e., drug: polymer, lipid concentrations and surfactant concentration 2:2, 10% and 1 mL, respectively.

### 2.2. Analysis of Optimization Data for the HLPHNs

The Design-Expert software (trial version 11, Stat-Ease Inc. Minneapolis, MN, USA) was utilized for the analysis of observed response and to develop all of the formulations used in this investigation. The following quadratic equation was generated for the optimization of HLPHNs concerning PS, %EE, and %DR:

$$\begin{array}{l} \%\;EE\;=\;+38.63\;+\;26.27\;\times \;X_{1}{+}_{}7.04\;\times \;X_{2}{+}_{}0.0000\;\times \;X_{3}-\;1.90\;\times \;X_{1}X_{2}{+}_{}0.0000\;\times \;X_{1}X_{3}-\;1.45\;\times \;X_{2}X_{3}-\;3.14\;\times \;X_{1}{}^{2}\\ +0.0000\;\times \;X_{2}{}^{2}{+}^{}0.0000\;\times \;X_{3}{}^{2} \end{array}$$

$$\begin{array}{l} \%\;DR\;=\;+18.87\;+\;30.68\;\times \;X_{1}{+}_{}6.45\;\times \;X_{2}{+}_{}0.0000\;\times \;X_{3}-\;0.8813\;\times \;X_{1}X_{2}{+}_{}0.0000\;\times \;X_{1}X_{3}-\;3.49\;\times \;X_{2}X_{3}-\;3.88\;\times \;X_{1}{}^{2}\\ +0.0000\;\times \;X_{2}{}^{2}{+}^{}0.0000\;\times \;X_{3}{}^{2} \end{array}$$

$$\begin{array}{l} PS\left(nn\right)\;=\;+8.85\;+\;8.67\;\times \;X_{1}{+}_{}34.96\;\times \;X_{2}{+}_{}0.0000\;\times \;X_{3}-\;6.37\;\times \;X_{1}X_{2}{+}_{}0.0000\;\times \;X_{1}X_{3}-\;15.14\;\times \;X_{2}X_{3}-\;1.01\;\times \;X_{1}{}^{2}\\ +0.0000\times X_{2}{}^{2}{+}^{}0.0000\times X_{3}{}^{2} \end{array}$$

**Figure 2:**
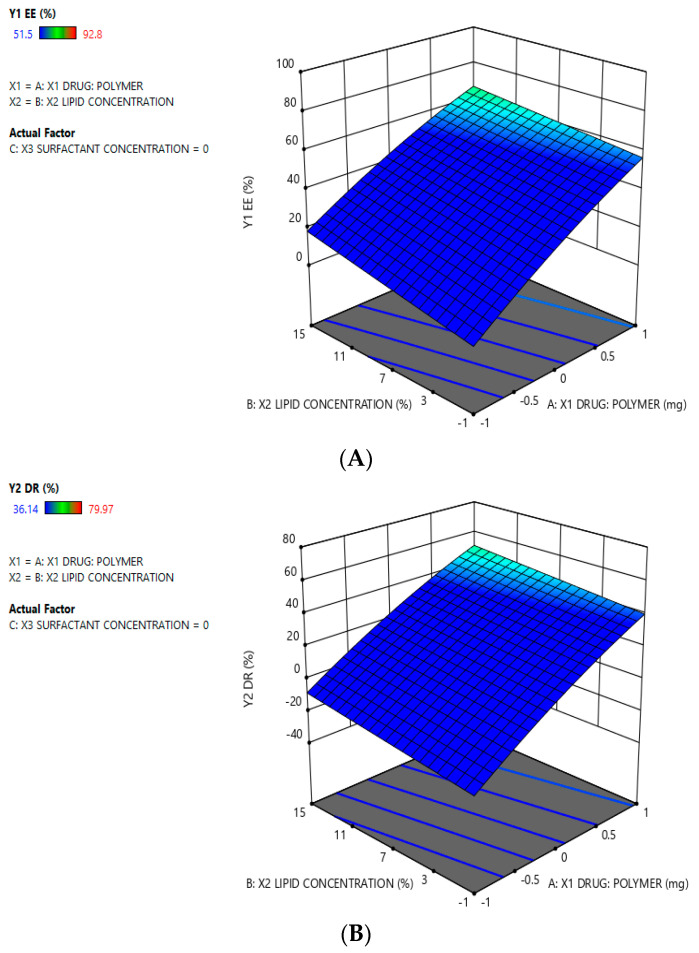

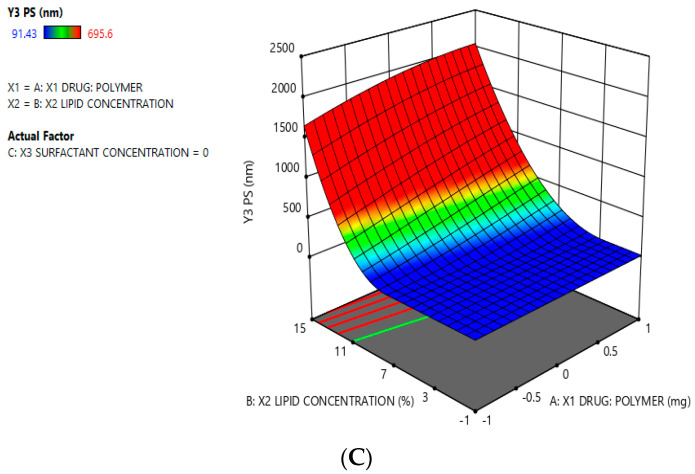
Effect of specified variables on hesperidin nanoformulations as depicted in three-dimensional response surface diagrams; (**A**)—%Entrapment efficiency of optimized formulation; (**B**)—%Drug release of optimized formulation; (**C**)—Particle size of optimized formulation.

The coded values of drug-to-polymer ratio, HLPHNS lipid concentration, and surfactant concentration were shown in panels X_1_, X_2_, and X_3_, respectively. A positive value factor of the equations means that the reaction was improved and vice versa. Table 1 shows all correlation coefficients (R^2^), standard deviation (SD), and results from ANOVA. The design was relevant to the measured response variables, as evidenced by the R^2^ value and the ANOVA results for the dependent variables. In addition, the quadratic equation suggests that an optimized formulation could be produced by mixing a specific ratio of lipids and polymers. It was reported that both the lipid and polymer had a remarkable effect on particle size, entrapment efficiency and drug release. The positive response was clearly seen in the surface graph in Figure 2. The results of PS, % EE, and % DR, obtained from these models are explained in the corresponding sections.

### 2.3. Screening HLHNPs Using Surface Methodology Using Box-Behnken DESIGN

The statistical analysis provided the desired amount of different ratios of drug polymer, surfactants and lipids to achieve the best results in terms of optimized formulation. Experimental design is an efficient statistical method to evaluate the influence of the independent variables on the dependent variables (in this case particle size, %DR and %EE). The physicochemical properties of the lipid nanoparticles were finally determined. The effect of the ratio of surfactants and lipids on HLHNPs was estimated using the solvent evaporation emulsion method. The result of the interaction between surfactant and lipid ratio (2:2) affected the particle size as a function of drug concentration. The HLPHNs were formulated using the optimal formulation variables, i.e., drugs: polymer, lipid concentrations, and surfactant concentrations of 2:2, 10%, and 1 mL, respectively, based on the squared reaction surface results. The predicted value of the formulation and the observed value of the formulation are shown in Table 2. The results showed that the optimized formulations were both appropriate and achieved the desired results. The response surface plot represents the relationship between the dependent and independent variables. Figure 2A–C show 3D response surface plots with the main statistical factors of the estimated parameters using Design-Expert software (trial version 11).

### 2.4. Particle Size, Zeta Potential and PDI Determination

**Figure 3:**
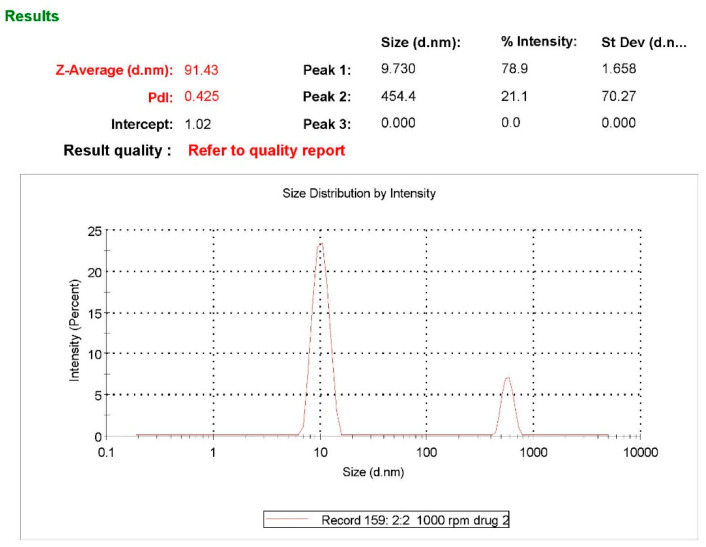
Zeta potential analysis of optimized hesperidin-loaded lipid polymer hybrid nanoparticles (HLPHNs).

The particle size of the formulation was determined using a particle size analyzer (Zetasizer S90, Malvern, Worcestershire WR14 1XZ, United Kingdom). For all HLPHN formulations, the particle size ranged from 91.43 to 695.6 nm and the zeta potential ranged from 0.0372 to 46.9, while the PDI ranged from 0.214 to 1.000 (Table 3 and Figure 3). The particle size, zeta potential and PDI data are necessary parameters that define the quality and stability of HLPHNs. The results show that variations in polymer concentration led to changes in nanoparticle size, PDI and zeta potential. Increased polymer concentration decreased nanoparticle size and contracted display HLPHN dispersions with the highest lipid content were larger compared to the largest particle size than dispersions with low lipid content. In addition, it was observed that the zeta potential also changed with increasing lipid content. The formulation with code HLPHN4 exhibited better particle size, zeta potential and PDI compared to the other formulations.

### 2.5. Entrapment Efficiency Percentage (%EE)

The EE improved with the increase in the loading capacity of the drug. This is because more lipid is required to encapsulate the high lipid concentration (LC) drug, resulting in an increased EE with a significant increase in particle size. A completely different behavior pattern was observed when LC was very low. However, the total amount of DL decreased significantly with the increase of LC. The increased ratio of hesperidin and polymer had a positive effect on % EE. The values for % EE of each formulation ranged from 51.5 to 92.8%. The % EE of drug HLPHN4 was compared with the other formulations. Based on the results of particle size, % EE, zeta potential and PDI determination, HLPHN4 was considered optimized.

### 2.6. Infrared (IR) Spectroscopic Analysis of HLPHNs

**Figure 4:**
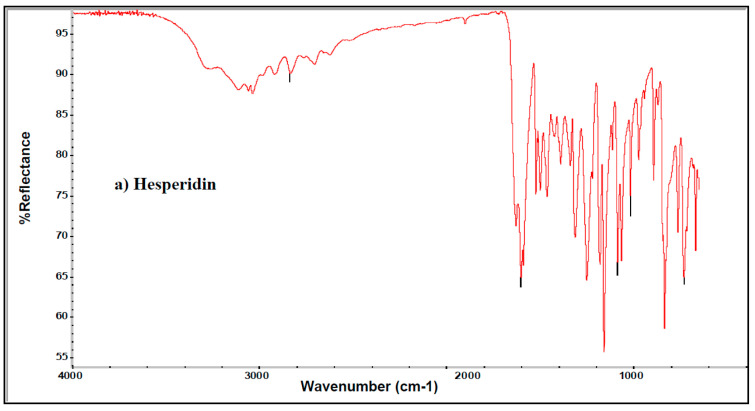

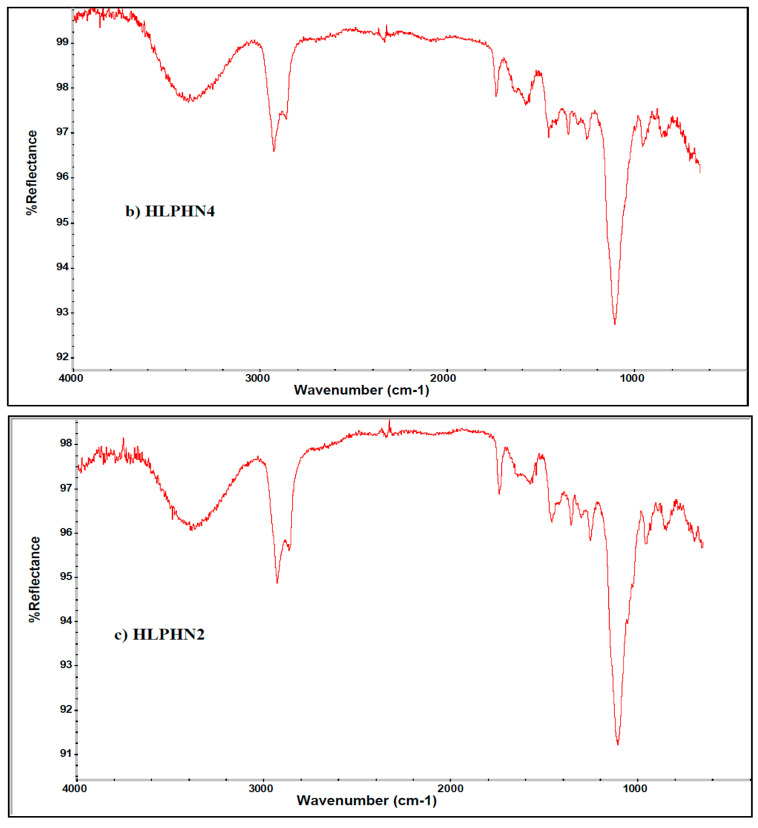
FTIR spectral analysis of (**a**) hesperidin (**b**) HLPHN4 (**c**) HLPHN2 under the optimized conditions.

The FTIR spectra of hesperidin and the optimized nanoparticles (Figure 4a–c) indicate a prominent characteristic peak between 3422.95 and 3544.61 cm^−1^, assigned to the stretching vibration frequency of the hydroxyl (–OH) group. The peak at 1648.88 indicates the presence of carbonyl (-C=O) functional groups. The peak between 1519.24 cm^−1^ and 1606.98 cm^−1^ represents the aromatic ring (-C=C- stretching). Continuous peaks were found between 1050.33 cm ^−1^ and 1300.94 cm ^−1^ due to ether linkage -C-O-C- and –C-O stretching. The peak between 970.39 cm^−1^ and 1034.08 cm^−1^ indicates deformation and rocking vibrations of -C-H. The continuous peak that appeared between 420.74 cm^−1^ and 466.70 cm^−1^ is due to out of plane and in-plane deformation of rings of hesperidin. The hesperidin spectra showed a characteristic peak at 2923.59 cm^−1^ which is due to the symmetric stretching of the sp^3^ hybridized carbon-hydrogen of soya lecithin, i.e., -CH_2_ and–CH_3_ symmetric stretching and asymmetric stretching. A prominent peak at 1736.54 cm^−1^ is assigned as a presence of ester functional group, i.e., soya lecithin ester group in nanoparticles in Figure 4b. The peaks between 1249.17 cm^−1^ and 1467.44 cm^−1^ are indicating the presence of –C-O-C- and –C-H groups respectively, because of stretching and bending of these groups respectively. A very sharp peak found to 1102.66 cm^−1^ is representing the attendance of esters –C-O- vibration in soya lecithin. The peak of 944.19 cm^−1^ and 962.13 cm^−1^ is due to the deformation of -C-H in nanoparticles Figure 4c. Hence the findings demonstrated that there were no substantial differences between the IR peaks of hesperidin and the optimized formulation indicating an interaction between the drug and polymer.

### 2.7. DSC Study

**Figure 5:**
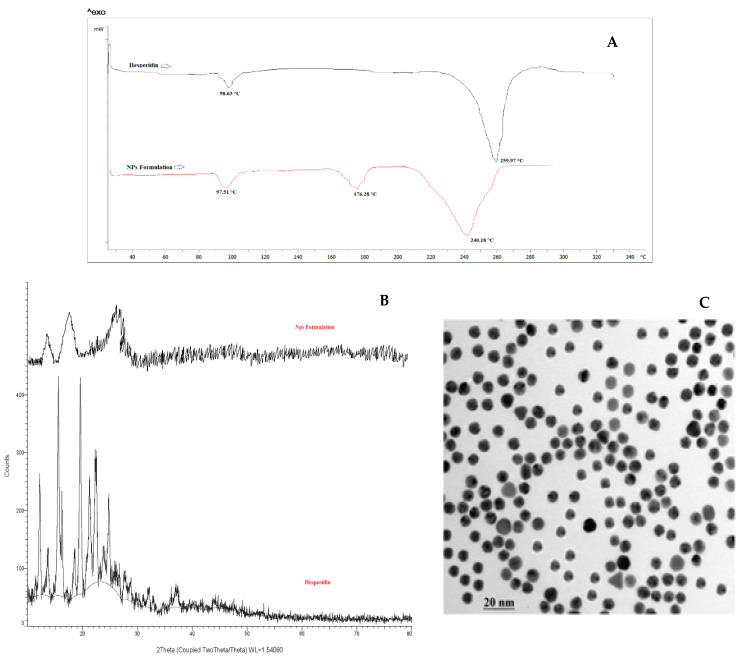
Hesperidin-loaded lipid polymer hybrid nanoparticle: (**A**)—DSC; (**B**)—XRD; (**C**)—TEM analysis.

The thermograms of hesperidin (pure drug) and HLPHNs are shown in Figure 5A. The DSC thermogram of hesperidin shows a modest peak at 98.63 °C and a sharp endothermic peak at 259.97 °C, indicating the melting of hesperidin and proving its crystalline state. The DSC thermogram of HLPHNs (endothermic peak at 240.18 °C) showed the absence of the endothermic melting peak of hesperidin, indicating that hesperidin was fully encapsulated in nanoparticles in an amorphous state, which exhibited different thermal properties than pure hesperidin. In addition, the area of the peak of HLPHNs increased, confirming the complete incorporation of hesperidin into the nanoparticles. The -OH group of hesperidin and the polar part of the lipid form hydrogen bonds that allow interaction between the lipid and hesperidin. The melting of the hesperidin-lipid complex is also responsible for the sharp peak in the HLPHNs curve at 176.28 °C [12,13,14]. As the temperature rises, hesperidin and lipid interact to form a complex that has a lower melting point than either of the individual components.

### 2.8. XRD Study

The most trusted technique to assess HLPHN formation was XRD. Sharp diffraction peaks were observed for hesperidin at 5.1, 8.5, 17.8, 19.6, 21.5, 22.3, and 24.8 (2 theta), while the HLPHN formulation showed peaks at 7.6, 15.8 and 26.8, respectively, in Figure 4b. The characteristic sharp peaks of hesperidin indicate the crystalline properties of pure hesperidin. The XRD diffractograms of the HLPHNs formulation show an altered intensity of some peaks as well as a broadening of the peaks, which could be due to the fact that the hesperidin forms a complex with the lipid in the nanoparticles. It was found that no characteristic peaks corresponding to pure hesperidin were observed in the HLPHN formulation. This indicates that the hesperidin in the nanoparticles is in amorphous form [15,16]. Moreover, a broadened peak of HLPHNs is seen, indicating the complete encapsulation of hesperidin in the nanoparticles. The DSC and XRD results prove that the hesperidin in the nanoparticles is amorphous in nature.

### 2.9. TEM Analysis of Modified HLPHNs

The TEM was also utilized to estimate nanostructure materials and size range of modified HLPHNs as sensing probe. The conclusions of the TEM photomicrograph are demonstrated in Figure 5C. It indicates that optimized HLPHN4 had a spherical shape with a particle size of about 500 nm. Therefore, TEM analysis confirmed the size and shape of modified HLPHN4.

### 2.10. Antioxidant Activity of the HLPHN4

The in vitro antioxidant activity of HLPHN4 showed a strong antioxidant potential with an IC_50_ of 69.49 µg/mL (Table 4). The oxidants known as reactive oxygen species (ROS) can cause or contribute to cellular damage. On the other hand, ROS may have beneficial effects on the body, particularly playing a critical role in the development of the normal wound healing response. Therefore, it is critical to maintain an appropriate balance between low and high levels of ROS. Although minimal amounts of ROS are beneficial for protecting tissues from infection and promoting successful wound healing by generating signals for cell survival. However, when high levels accumulate, ROS causes oxidative stress that leads to cellular damage and a pro-inflammatory state. New treatments are being developed that target antioxidant wound dressings to help maintain this delicate balance [17,18,19]. Topical administration of natural antioxidants is becoming increasingly popular because it allows more precise targeting of the upper layer of the skin, which is particularly beneficial. Because of their high molecular weight, unstable nature, limited absorption, susceptibility to degradation, and rapid systemic excretion, natural antioxidants delivered topically to the skin present a challenging research problem. In numerous cases, nanocarriers have been shown to improve the clinical efficacy and effectiveness of these drugs [20,21]. Merrell and colleagues demonstrated that nanofibers loaded with curcumin increased the rate of wound closure in a diabetic animal model with prolonged delivery of the drug. The formulation had a more uniform diameter distribution, with fibres ranging in size from 200 to 1000 nm [22]. By using gelatin-based electrospun nanofiber mats, Dai et al. were able to optimise a delivery system that increased the solubility and bioavailability of curcumin. In a rat model of acute wounds, application of these nanofiber mats accelerated the healing process, suggesting that this traditional drug could be used in modern wound care [21]. As a topical delivery method, quercetin was loaded onto lecithin-chitosan nanoparticles. Compared to pure quercetin, the quercetin-loaded nanoparticles showed better penetration and significant deposition of quercetin in the skin [23].

The biocompatibility, hydration, hemostasis, and anti-inflammatory properties of chitosan are well known in wound therapy, where it has been approved by the FDA. In addition, chitosan has the ability to accelerate cell proliferation and histoarchitectural tissue organization. It is bacteriostatic and fungistatic and facilitates rapid regeneration and re-epithelialization of the skin, thus acting as a wound healing accelerator [24]. The chitosan was used in the preparation of HLPHN4 and can produce synergistic wound healing properties with hesperidin. Chitosan was used in the preparation of HLPHN4, which can exert synergistic wound healing properties in combination with hesperidin. On the basis of researcher’s statement, the HLPHN4 having strong antioxidant activity and might produces higher healing efficacy, improved penetration rate, sustained release rate as well as better bioavailability. Hence, in future in vivo wound healing activity will be carried out for the validation of the healing efficiency of the optimized formulation.

### 2.11. In-Vitro Drug Release (DR) Studies

**Figure 6:**
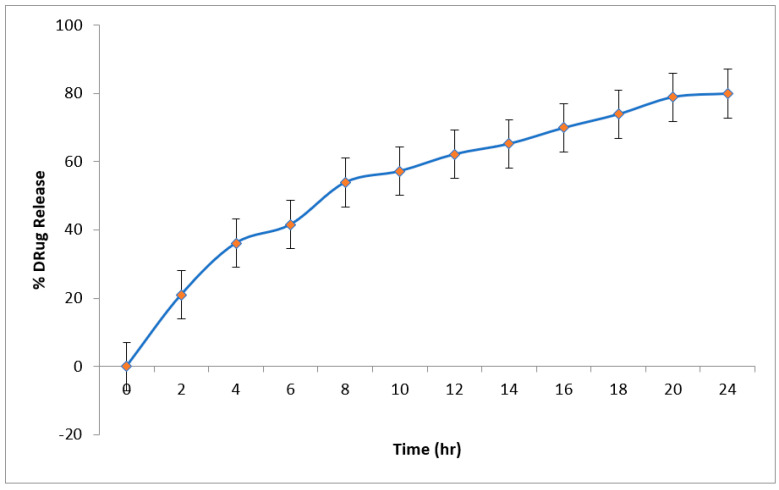
Drug release of hesperidin-loaded lipid polymer hybrid nanoparticles.

Figure 6 shows the cumulative percentage DR of the optimized HLPHN4 dispersion, which is 79.97% in 24 h. During the first 4 h, the explosive release of the drug from HLPHNs was observed. After 4 h, the release of drug from HLPHN4 was inhibited, indicating a delayed release of HLPHN4. The accessibility of the free hesperidin drug in the outermost layer, the lipid phase, could explain the burst release of the drug from HLPHN4. The sustained release of the drug due to the affinity between lipid drug and HLPHN4 suggests that the release of the drug from the inner polymer phase is delayed after the initial burst of release. The above discoveries showed that a moderate arrival of hesperidin from a pad with a pH of 6.8 could ensure that a sufficiently high centralization of HLPHN4 is taken up by the skin. The above results indicate that a slow release of hesperidin from the pH 6.8 buffer could ensure that a sufficiently high concentration of HLPHN4 could be taken up by the skin. Therefore, HLPHN4 may potentially provide a sustained release and be an effective delivery system for the skin.

### 2.12. Storage Stability Studies

The optimized HLPHN4 was kept at 4 °C and 25 °C for 3 months and its particle size, zeta potential and percentage EE were studied (Figure 6). The results of the stability study showed negligible increase in the particle size of the drug of the optimized HLPHN4. No changes in the zeta potential of the optimized HLPHN4 were observed during the three-month stability study. No changes were also observed in the percentage EE of the optimized HLPHN4, indicating that the drug can be retained in the HLPHN4 for a sufficient period of time. The results of the stability studies indicate that the optimized HLPHN4 are stable at 4 °C and 25 °C for a total period of 3 months. Nanoformulation stability is an important parameter in evaluating the clinical potential of HLPHN4. It indicates that the HLPHN4 is stable and safe to use.

## 3. Materials and Method

### 3.1. Chemical, Reagents and Solution Preparation

Hesperidin, poly vinyl alcohol (PVA), chitosan and soya lecithin were acquired from Hi-media (Mumbai, India). All other chemicals used in experiments were of analytical grade.

### 3.2. Development of Hesperidin Loaded Lipid-Polymer Hybrid Nanoparticles (HLPHNs)

The drug lipid-polymer hybrid nanoparticles were prepared using the emulsification solvent evaporation method following the proven methodology [25]. Soya lecithin and chitosan were dissolved in a beaker in 5 mL of DCM. Separately, 10 mg of hesperidin were dissolved in 0.3 mL of pH 6.8 phosphate buffer. The buffered drug solution was added to the previously prepared lipid polymer (oil) phase. After that 20 mL of PVA (1.5% w/v) solution was used as an aqueous phase to play role of stabilizing agent in the preparation of lipid nanoparticles [26]. In the next step, under high-speed stirring, the lipid component was added dropwise to the aqueous phase over 24 h. The solvent-containing organic phase (DCM) was completely evaporated under continuous stirring at room temperature. Every 5 min of the cycle, the dispersion of nanoparticles was sonicated with a bath sonicator, with a 3-min interval between each sonication cycle to avoid extreme heat generation and product degradation. Hesperidin-containing lipid-polymer hybrid nanoparticles were centrifuged at 14,000 rpm for 30 min. The supernatant of the prepared nanoparticles was discarded, and the solid mass was distilled three times before dispersing in Millipore water. The HLPHNs were lyophilized and stored in a hermetically sealed container for subsequent experiments, and details of the composition can be found in Table 4 [27,28].

### 3.3. Optimization of Modified HLPHNs

The various parameters used to optimize the HLPHN formulations, such as variable drug-polymer (X_1_), lipid concentration (X_2_), and surfactant concentration (X_3_), were determined through research. The result was that the thickness and consistency of HLPHNs depended on the lipid concentration, and 10% lipid was found to be the optimal concentration. Using the above observation, the value of entrapment efficiency (EE) was integrated for the optimized formulation. Experimental design techniques were used to create a suitable batch to study the interactions of hesperidin with lipids and their effects on the entrapment efficiency and particle size of the optimized formulation. The seventeen trials with odd HLPHNs were prepared using the emulsification solvent evaporation method under the experimental design, and the details are shown in Table 3, Table 5 and Table 6.

### 3.4. Surface Methodology Using the Box-Behnken Design: Statistical Parameters

The variables were examined and the sensitivity of responses to changes in experimental design settings was investigated using a Box-Behnken design with dependent and independent variables. As a result, a three-factor Box-Behnken design was selected. A numerical and statistical method that summarizes the functional relationship between different ratios was used to determine an optimal response. The effect of different dependent variables characterized by percent particle size, percent drug release, and percent entrapment efficiency of the prepared nanoparticles was presented using three-level factorial designs. Various parameters, such as the drug to polymer ratio (X_1_), lipid concentration (X_2_), and surfactant (X_3_), were selected. The significant best-fit model was determined by statistical analysis with a a *p* 0.05 value. The R^2^ value was assumed, and an analysis of variance (ANOVA) was used to ensure that the model fit was perfect. Box-Behnken (3D) response surface curves [29] were used to investigate the interaction between independent and dependent variables. Comparisons between experimental and predicted values are used to evaluate the reliability of the model, and the result is expressed as a percent bias. The following equation was used to calculate the bias value:

$$\%\mathrm{Bias}\;=\frac{\mathrm{predicted}\;\mathrm{value}-\mathrm{experimental}\;\mathrm{value}}{\mathrm{predicted}\;\mathrm{value}}\times 100$$

Table 1 shows that the formulation procedure was optimized by looking at several formulation factors such drug polymer ratio (X_1_), lipid concentration (X_2_), and surfactant concentration (X_3_).

### 3.5. Characterization of Modified HLPHNs

#### 3.5.1. Particle Size, Zeta Potential, and PDI Determination

A Malvern Zetasizer S90 (Malvern Instruments Ltd., Worcestershire WR14 1XZ, UK) particle size analyzer was used to investigate the particle size of the formulations. The solutions were sonicated and measure the particle size after dilution, its measurement shown in Table 2. Stability of HLPHNs were determined through various parameters such as mean vesicle size, size distribution and zeta potential [30].

#### 3.5.2. Fourier Transform Infrared Spectroscopy (FTIR) Analysis of LPHNPs

FTIR enables the analysis of interfaces to study the surface adsorption of functional groups in the nanoparticles [31]. An FTIR spectrometer (FTIR alpha model, Bruker, Billerica, MA, USA) and the KBr pelletization technique were used to obtain the absorption spectra. The homogeneous disc was compacted in a palletized form and carefully placed in an IR sample holder, and the results were recorded for future use.

#### 3.5.3. Differential Scanning Calorimetry (DSC)

DSC (DSC 1 STAR system, Mettler Toledo, Greifensee, Swizerland) thermograms of the samples were recorded. The interactions between hesperidin and polymers were studied using DSC. Throughout the processing, it is used to evaluate the possibility of any form of interaction between the phospholipids and HLPHNs. The 5 mg lyophilized HLPHNs and hesperidin were kept separately in the pan. The pan was heated to 300 °C at a rate of 10 °C/min, with N_2_ gas pumped into the chamber at a rate of 20 mL/min. A thermogram curve was prepared for the formulation of HLPHNs [32].

#### 3.5.4. X-ray Diffraction (XRD) Analysis

To screen and characterize the nature of HLPHNs, XRD (D2 PHASER, Bruker, Billerica, MA, USA) was used. Using powder X-ray diffractometry, researchers investigated the X-ray powder diffraction of pure hesperidin and lyophilized HLPHNs. samples were scanned from 2^θ^ to 90^θ^ at a voltage of 40 kV and a current of 30 mA [33].

#### 3.5.5. Transmissions Electron Microscopy (TEM) Study

The morphology of HLPHNs, their size, and surface area were assessed using TEM (TALOS, Thermo fisher scientific India Private limited, Mumbai, India). An optimized batch was evaluated using the negative staining technique. The HLPHNs were used in aqueous dispersion on copper grids. Samples were dried and properties were examined under a microscope at 10–100× magnification and magnified up to ×150,000 [34].

### 3.6. Antioxidant Activity of the HLPHNs

The DPPH radical scavenging activity of HLPHNs was used to assess their antioxidant activity. The colloidal HLPHNs solution at different concentrations from 50–250 µg/mL was added to 2.95 mL of ethanol solution containing 0.1 nM DPPH. Then the samples were shaken and incubated in the dark at a temperature of 25 °C for 90 min. The corresponding amount of samples was scanned by an UV spectrophotometer (Shimadzu, Toshvin Analytical Pvt. Ltd., Mumbai, India) at 520 nm using ethanol as blank solution. The DPPH inhibition capacity of the sample was calculated using the following equation. [35].

% AA = 100 − {[(Abs_sample_ − Abs_blank_) × 100]/Abs_DPPH_}

### 3.7. Entrapment Efficiency Percentage (%EE)

The prepared formulations were centrifuged for 30 min at 14,000 rpm at 40 °C. UV spectrophotometers were used to analyse the completely clear effluent at 285 nm, and the concentration of the (percent EE) was calculated employing the formula below [36].

$$\%\;\mathrm{Entrapment}\;\mathrm{efficiency}=\frac{\mathrm{Drug}\;\mathrm{added}\;-\;\mathrm{unetrapped}\;\mathrm{drug}\;}{\mathrm{Drug}\;\mathrm{added}}\times 100$$

### 3.8. In-Vitro Drug Release from HLPHNs as Sensing Probe

The diffusion cell assembly using an egg membrane was used to study the release of hesperidin from optimized HLPHNs formulations. The formulations were kept in the donor compartment with phosphate buffers with a pH of 6.8 at a temperature of 35 ± 0.5 °C. The aliquots were withdrawn from the compartment at different intervals such as 0, 0.25, 0.5, 1, 2, 3, 4, 6, 8, 10, 12, 14, 16, 18, 20, 22 and 24 h and sink conditions were maintained. UV spectroscopy (Shimadzu 1800) at a wavelength of 285 nm was used to determine the amount of hesperidin in the test sample [37].

### 3.9. Storage Stability Studies

A 10 mL solution with a drug concentration of 2 mg/mL was stored at 4 °C and 25 °C for three months to assess the stability of the optimized HLPHNs. At a sampling frequency of three months, the stability test was analyzed using PS and determination of dispersion in % EE [30,38,39].

## 4. Conclusions

The use of HLPHNs has demonstrated a wide range of success in the implementation of novel clinical and drug delivery applications, as demonstrated by our recent evaluation of a novel drug delivery system for in vitro antioxidant activity in the DPPH model. The results of the current study suggest that the interaction between the ratio of surfactants and lipids affects particle size or decreases particle size as a function of drug concentration. RSM was used to optimize the HLPHNs produced by fitting a second order model to the reaction data. HLPHN4 showed the best results in terms of particle size distribution, zeta potential, drug release and entrapment efficiency compared to the other formulations and was therefore considered as the optimized formulation. The positive DSC and XRD results showed that the physical stability of the loaded drug in a hybrid system with a complete absence of peaks suggesting the physical stability of the hesperidin-lipid complex in the optimized formulation. In vitro drug release of HLPHN4 confirmed sustained release and the most effective delivery system for skin. The present study suggests that HLPHN4 is stable and could also improve the topical bioavailability of hesperidin due to its nano size with larger surface area.

In conclusion, we demonstrate that hesperidin-loaded lipid-polymer hybrid nanoparticles can serve as a suitable transporter for the delivery of bioactive hesperidin to wounds, leading to improved wound healing. In addition, the optimised formulation needs to be studied in vivo in the future to support the current findings.

## Figures and Tables

**Table 1:** Details of response regression and analysis of variance for various parameters.

Variables	df	SS	Ms	F	*p*	R^2^	SD
%EE							
Model	5	2262.16	452.43	16.56	<0.0001	0.8827	5.23
Residual	11	300.59	27.33	-	-	-	-
Total	16	2562.74	-	-	-	-	-
%DR							
Model	5	2799.73	559.95	20.30	<0.0001	0.9022	5.25
Residual	11	303.37	27.58	-	-	-	-
Total	16	3103.10	-	-	-	-	-
PS (nm)							
Model	5	339.63	67.93	3.98	0.6262	0.6442	4.13
Residual	11	187.60	17.05	-	-	-	-
Total	16	527.23	-	-	-	-	-

Where, EE—Entrapment efficiency; DR—Drug release; PS—particle size; SD—Standard deviation; SS—Sum of square; MS—Mean of square.

**Table 2:** Data of predicted and observed values for the HLPHNs.

Variable	Predicted Value	Observed Value	Bias Percentage
%DR	65.94	60.95	7.34
%EE	68.8	72.36	−5.17
PS (nm)	73.32	80.10	−9.24

**Table 3:** Data of EE, DR, PS, PDI and Zeta Potential of various batches of HLPHNs.

Formulation Code	Coded level			Actual Level			%EE	%DR	PS(nm)	PDI	Zeta Potential (mv)
	X_1_	X_2_	X_3_	X_1_	X_2_	X_3_					
HLPHN 1	−1	0	−1	1:1	10	0.5	91	75.96	695.6	0.026	16.6
HLPHN 2	+1	0	+1	2:3	10	1.5	58.9	41.6	490.8	0.071	−0.0771
HLPHN 3	−1	−1	0	1:1	5	1	70	77.96	695.6	0.043	37.9
HLPHN 4	0	0	0	1.5:2	10	1	92.8	53.93	91.43	0.056	15.6
HLPHN 5	0	0	0	1.5:2	10	1	92.6	62.2	143.1	0.036	0.242
HLPHN 6	+1	0	−1	2:3	10	0.5	51.5	36.14	158.4	0.029	46.9
HLPHN 7	0	0	0	1.5:2	10	1	91.8	57.25	158.1	0.098	37.5
HLPHN 8	−1	−1	0	1:1	5	1	72.8	78.96	672.6	0.362	37.5
HLPHN 9	0	0	0	1.5:2	10	1	78.2	65.25	649	0.348	−0.0372
HLPHN 10	0	−1	+1	1.5:2	5	1.5	82.4	69.94	390.9	0.650	38.2
HLPHN 11	0	+1	−1	1.5:2	15	0.5	88	73.94	551.2	0.257	4.82
HLPHN12	−1	0	−1	1:1	10	0.5	76	79.97	675.3	0.342	6.83
HLPHN 13	+1	+1	0	2:3	15	1	65.9	46.43	494.3	0.453	0.242
HLPHN 14	0	+1	+1	1:1	15	1.5	85.5	72.94	556.4	0.619	32.3
HLPHN 15	0	0	+1	1:1	10	1.5	90.5	74.95	670.1	0.552	34.1
HLPHN 16	+1	−1	0	2:3	5	1	67	49.89	468.6	0.196	4.82
HLPHN 17	0	−1	−1	1.5:2	5	0.5	82.1	67.94	538.1	0.369	−0.0372

**Table 4:** Antioxidant activity of HLPHN4.

Concentration (µg/mL)	DPPH Scavenging %
50	46.2 ± 0.14
100	63.7 ± 0.29
150	98.1 ± 0.07
200	121.4 ± 0.56
250	153.6 ± 0.33
IC_50_	64.49 (µg/mL)

Values are mean ± SEM of six determinations.

**Table 5:** Different components employed in formulation of HLPHNs.

Independent Variable	−1 (Low)	0 (Medium)	+1 (High)
Drug: polymer (mg) (X_1_)	1:1	1.5:2	2:3
Lipid concentration (%) (X_2_)	5	10	15
Surfactant concentration (ml) (X_3_)	0.5	1	1.5

**Table 6:** Composition of HLPHNs.

Formulation Code	Amount of Drug (Hesperidin) (mg)	Amount of Polymer (Chitosan) (mg)	Amount of Lipid (Soya Lecithin) (mg)	Amount of Surfactant (mL)
HLPHN 1	10	10	10	0.5
HLPHN 2	20	30	10	1.5
HLPHN 3	10	10	5	1
HLPHN 4	15	20	10	1
HLPHN 5	15	20	10	1
HLPHN 6	20	30	10	0.5
HLPHN 7	15	20	10	1
HLPHN 8	10	10	5	1
HLPHN 9	15	20	10	1
HLPHN 10	15	20	5	1.5
HLPHN 11	15	20	15	0.5
HLPHN12	10	10	10	0.5
HLPHN 13	20	30	15	1
HLPHN 14	10	10	15	1.5
HLPHN 15	10	10	10	1.5
HLPHN 16	20	30	5	1
HLPHN 17	15	20	5	0.5

## Data Availability

Data is contained within the article.
